# Contextual influences in the peripheral retina of patients with macular degeneration

**DOI:** 10.1038/s41598-019-45648-4

**Published:** 2019-06-26

**Authors:** Giulio Contemori, Luca Battaglini, Clara Casco

**Affiliations:** 1 Department of General Psychology, University of Padova, Via Venezia 8, 35131 Padova, Italy; 2 Neuro.Vis.U.S. Laboratory, University of Padova, Padova, Italy; 3 Université de Toulouse-UPS, Centre de Recherche Cerveau et Cognition, Toulouse, France

**Keywords:** Visual system, Retina

## Abstract

Macular degeneration (MD) is the leading cause of low vision in the elderly population worldwide. In case of complete bilateral loss of central vision, MD patients start to show a preferred retinal region for fixation (PRL). Previous literature has reported functional changes that are connected with the emergence of the PRL. In this paper, we question whether the PRL undergoes a use-dependent cortical reorganization that alters the range of spatial lateral interactions between low-level filters. We asked whether there is a modulation of the excitatory/inhibitory lateral interactions or whether contextual influences are well accounted for by the same law that describes the integration response in normal viewers. In a group of 13 MD patients and 7 age-matched controls, we probed contextual influences by measuring the contrast threshold for a vertical target Gabor, flanked by two collinear high-contrast Gabors. Contextual influences of the collinear flankers were indicated by the changes in contrast threshold obtained at different target-to-flanker distances (λs) relative to the baseline orthogonal condition. Results showed that MDs had higher thresholds in the baseline condition and functional impairment in the identification tasks. Moreover, at the shortest λ, we found facilitatory rather than inhibitory contextual influence. No difference was found between the PRL and a symmetrical retinal position (non-PRL). By pulling together data from MD and controls we showed that in the periphery this inversion occurs when the target threshold approach the flankers’ contrast (about 1:3 ratio) and that for patients it does occur in both the PRL and a symmetrical retinal position (non-PRL). We conclude that contrary to previous interpretations, this modulation doesn’t seem to reflect use-dependent cortical reorganization but rather, it might result from a reduction of contrast gain for the target that promotes target-flankers grouping.

## Introduction

Macular degeneration (MD) causes loss of input to the region of the primary visual cortex that represents the fovea. The evidence that the adult brain is capable of plasticity^1–3^ raises the question of how the cortical deafferentation in MD affects cortical functionality and, in particular, whether cortical rearrangement occurs.

The cortical reorganization hypothesis is compatible with the results of the animal studies reporting the expansion of receptive fields of neurons near the retinal lesion boundary^3–10^. fMRI studies in humans provide instead conflicting evidence in support of the plasticity hypothesis. Some found clear activation of the foveal cortex to stimuli presented outside the central scotoma^11–15^, while others did not show evidence of this functional reorganization^16–19^. Some of the behavioral changes in MDs support, but only indirectly, the cortical reorganization hypothesis. For example, Safran and Landis^20,21^ reported that MD patients, similarly to what was found with artificial scotoma^22^, experienced apparent displacement of images adjacent to the scotoma toward the field defect leading to perceptual completion and shape distortion. De Stefani and colleagues^23^ demonstrated perfect discrimination of the curvature of illusory contours across the pathological scotoma. They suggested that, following the loss of bottom-up input, the visual cortex enhances connectivity and/or low-spatial frequency response, thus mediating the formation of a neural representation of complex geometrical shapes across the scotoma. In the preferred retinal locus (PRL) of the MD patients, Chung^24^ showed a “foveal-like” distortion of the scaling of critical spacing with the eccentricity. Casco and coworkers^25^ provided evidence that MD patients are better than normally sighted observers in using information allowing detection of the mirror, but not the translational symmetry of a two-dot configuration at the opposite side of the scotoma. This last result is compatible with cortical rewiring, whereby detection of the co-aligned low spatial filters crossing the scotoma becomes more efficient with MD. However, a more parsimonious explanation is that MD’s peripheral vision takes its functional advantage from more efficient use of the high-level representation of the visual input. Indeed, the numerous functional changes that have been observed in MD vision^20,24–26^ are compatible with the suggestion that the visual response relies more on an integration field output.

To summarize, there is an important question that have no answer yet. Do the functional changes observed in the PRL result from modulation of excitatory and inhibitory contextual influences involved in contrast detection? To answer this question in this work we have looked at whether MD’s peripheral vision is associated with a change in contextual influences with respect to controls and whether this change is accounted for by the same model that describes the contrast gain in normal viewer^27^. There are different ways in which the observed pattern of contextual influences in the periphery might be different in MD with respect to controls. In people with normal vision, accumulating psychophysical studies have shown contextual influences on the threshold for contrast detection coming from outside the receptive field of the channel responding to the target^1,27–29^. In particular, it is well established that the visibility of a low contrast Gabor patch is affected by collinear flanking Gabors of similar orientation and spatial frequencies but high contrast: short target-to-flanker separation (1–2 times the wavelength of the target Gabor’s carrier, λ) leads to suppression, whereas target-to-flanker separations of 3-4λ lead to enhancement^27–33^. Moreover, these contextual effects can be modulated by task repetition in a perceptual learning paradigm, in both normally sighted observers^33,34^ and patients with impaired vision^35–43^. In addition, contextual influences depend on eccentricity: in the periphery, inhibition is more prominent and contextual enhancement occurs at a target-to-flanker distance of 6λ, that is larger than in the fovea^32,34,44^.

Since lateral connectivity has been shown to increase with practice in normal^31,34,45^ and pathological vision^46–48^, use-dependent cortical reorganization might change the observed contextual influences. A partial support for the cortical reorganization hypothesis comes from recent papers showing that MD patients exhibited a modulation of contextual influences in respect of controls^35,49^. In the present study, we further address this issue attempting to specify the underlying mechanism. In addition, by comparing the effect of flankers distance on contrast detection both in the PRL and in a symmetrical retinal position (non-PRL) we also hope to address the still debated issue of whether the vision in the PRL is enhanced by the use of this region for everyday visual tasks^13,15,50,51^. A contextual modulations for target contrast detection with specific properties in the PRL would support the “Use-Dependent Reorganization” hypothesis while a strong similarity between the two tested locations would play in favour of a more conservative “Use-Independent Reorganization” hypothesis^13^. Finally, we asked whether a modulation of contextual influences in MDs affects the efficiency in performing everyday visual tasks.

## Methods

### Participants

Participants were 13 MD patients (mean age of 61 ± *9.16* years, range: 49–83 years) and 7 controls (mean age of 59 ± *4.12* years, range: 54–64 years). Patients were selected based on clinical history and Nidek MP1 microperimetry results. The dispersion of fixation was quantified during the microperimetry, and only patients with at least 80% of fixations in the range of 2° of visual angle around the focal point of the PRL were included in the sample. Patients with concomitant visual diseases other than central vision loss were not included, nor were those with a visual acuity on the ETDRS eye-chart lower than 1/20 or above 15/20. All the tests were performed monocularly and, although patients had a bilateral scotoma, the chosen eye was the one with the best-spared vision based on visual acuity and microperimetry data. The eye chosen to be tested for the control group was the non-dominant eye. Because the non-PRL was defined as the symmetrical retinal location of the PRL, the proximity of the optic disc or the irregular shape of the scotoma could reduce the visibility of stimuli presented in this second retinal location. To check for visibility of the stimuli, during both the crowding and lateral masking tasks, patients were asked to report the number of visible elements in the non-PRL. Of the 13 MD patients, all could see the full triplet of stimuli (Gabors and letters) in the PRL, while only 8 of them could do so in the non-PRL. Because the presentation of the stimuli was randomized in the two retinal positions to reduce the frequency and the amplitude of eventual eye movements, all the patients were tested in both locations, but only the ones who were able to see the full triplets were further considered for statistical analysis in the non-PRL position.

Details relative to age, gender, scotoma diameter, visual acuity, and PRL position are summarized in Table 1. The study was performed in accordance with the ethical standards laid down by the Declaration of Helsinki^52^. The study was approved by the Ethics Committee of the Department of General Psychology, University of Padova (Protocol 1449). We obtained written informed consent from all participants involved in the study.

**Table 1:** Details of participants. The MD group consisted of six patients with AMD, one patient with juvenile macular degeneration (JMD), two patients with Stargardt disease, one with Best disease, one with a macular hole, one with cone-rod dystrophy (CRD), and one with central serous chorioretinopathy.

Patients	Deficit	Gender	Age	Scotoma size (diameter)	Position of PRL	Tested eye	(VA)
MD1	CRSC	Male	50	4°	Left-up 2.0°-1.0°	LE	2/10
MD2	Macular hole	Female	49	3°	Right-up 1.5°-1.0°	RE	7/10 (LAC)
MD3	Best disease	Male	58	8°	Left-up 4°-2.7°	LE	2/10
MD4	CRD	Male	62	6°	Left 4.5°	RE	2/10
MD5	Stargardt	Male	69	7°	Left-down 2.5°-6	LE	1/10
MD6	JMD	Female	56	4°	Right-up 2°-1°	RE	2/10
MD7	AMD	Female	62	3	Right-down 1°-1°	LE	2/10
MD8	AMD	Female	65	3°	Left 1°.5	RE	2/10
MD9	AMD	Female	63	6°	Left-down 4.5°-2°	LE	2/10
MD10	AMD	Male	61	10	Left-down 8°-1°	LE	3/10
MD11	AMD	Male	65	6°	Left-up 4°-2°	RE	1/10
MD12	Stargardt	Male	50	10°	Right-up 6°-6.5°	RE	1/10
MD13	AMD	Male	83	9°	Left-down 2°-2°	LE	3/10
C1	none	Male	58	none	none	Non-dominant	10/10
C2	none	Female	64	none	none	Non-dominant	10/10
C3	none	Male	60	none	none	Non-dominant	10/10
C4	none	Female	59	none	none	Non-dominant	10/10
C5	none	Male	64	none	none	Non-dominant	10/10
C6	none	Male	54	none	none	Non-dominant	10/10
C7	none	Female	54	none	none	Non-dominant	10/10

The patients underlined are those considered in the non-PRL analyzes.

### Locations tested

**Figure 1:**
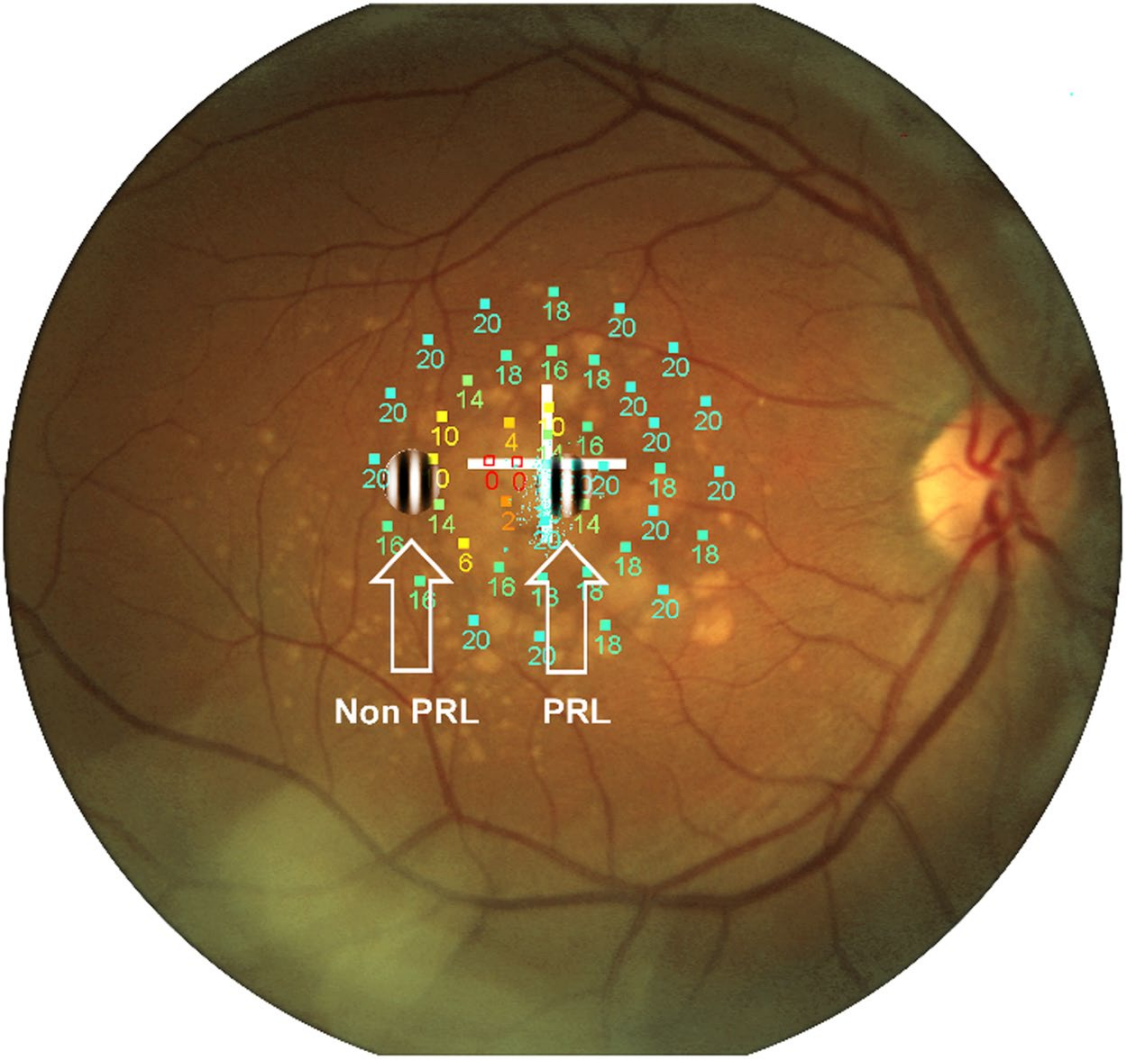
Illustrative example of stimuli placement. Stimuli presented at the PRL and at the non-PRL are superimposed over the microperimetry.

The eccentricity of the PRL was individually estimated as a proportion of the distance from the macula and the optic disk, in degrees of visual angle. Using the image of the retinal fundus, the position of the fovea was computed based on the averages of the values determined for normally sighted observers: 15.3° temporally and 1.5° below the center of the optic disc. The distance between the position of the fovea and the PRL position estimated by the microperimetry was then computed to derive the eccentricity of the PRL. The non-PRL position was defined as approximately correspondent to the horizontally specular area of the retina, using the macula as the center of symmetry. One example of patients’ microperimetry and the respective retinal displacement of the target Gabor in either the PRL or the non-PRL is shown in Fig. 1. In the case of very small scotoma, the non-PRL position was set by default at 6° of eccentricity with respect to the PRL on the horizontal axis. This was done to allow reliable discrimination between the two gaze positions by the eye tracker. The eccentricity at which stimuli were presented to each control subject matched that of one patient, randomly chosen. On average, the eccentricity was 4°.

### Eye movement recording

Participants’ fixation was controlled with an eye tracker to determine the retinal position corresponding to the patients’ PRL/non-PRL and to be sure that fixation was maintained. Calibration and recording procedures were as follows. Eye movements were recorded using a Mirametrix S2 eye tracker with a sample rate of 60 Hz and an accuracy of 0.5°. The calibration of the tracker and the gaze check were integrated into the main Matlab script using the Mirametrix Matlab Toolbox and API for Windows. Thanks to a custom calibration software, the calibration dot that normally sighted observers follow with the fovea was shifted by a constant so that, although MD patients could follow it with their PRL, the position of the eye relative to the calibration dot corresponded to that of a normally sighted observer. Due to the instability of fixation and the systematic error of the tracker, a tolerance window of ±1.5° around the PRL fixation point was set. If the gaze of the subject before the stimulus presentation was out of this window, a warning sound was presented to allow the patient to relocate his or her gaze.

### Apparatus and stimuli

Participants sat in a dark room 57 cm from the screen. Stimuli were displayed on an ASUS ML228H LCD LED 21.5-inch monitor with a refresh rate of 60 Hz and a spatial resolution of 1920 × 1080 pixels, with a pixel pitch of 0.248 mm. Stimuli were generated with Matlab Psychtoolbox^53,54^. Gamma correction for each color channel was applied through calibration with the Spyder 4 Elite colorimeter (DataColor). The calibration was further verified using a Minolta LS-100 photometer, which indicated that the mean luminance was 50 cd/m2. In that way, luminance was a linear function of the digital representation of the image.

In order to represent 10.7 bits of luminance (1786 gray levels) on an 8-bit display, we adopted a software solution called “Pseudo-Gray,” also known as “Bit-Stealing”^55^, implemented via a Psychtoolbox built-in function.

#### Contrast detection stimuli

**Figure 2:**
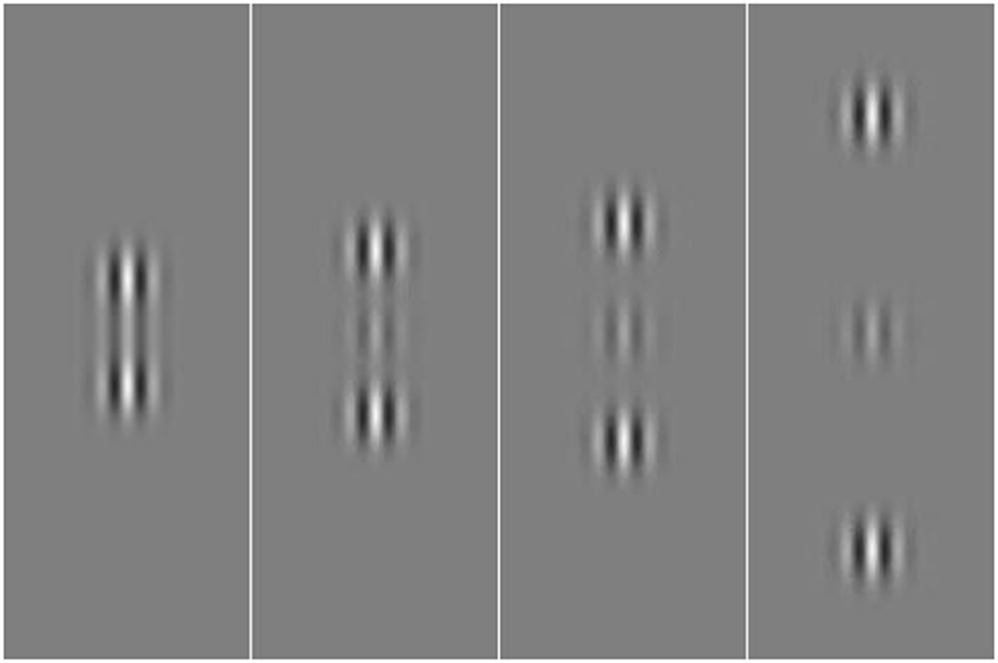
Stimuli used for the lateral masking paradigm. Increasing target-to-flanker separations of 2λ, 3λ, 4λ, and 8λ are shown.

Stimuli were Gabor patches consisting of a cosinusoidal carrier enveloped by a stationary Gaussian. Each Gabor patch was characterized by its sinusoidal wavelength (λ), phase (*φ*), and standard deviation of the luminance Gaussian envelope (⌠) in the (x,y) space of the image:

1 $$G(x,y)=\,\cos (\frac{2\pi }{\lambda }x+\phi ){e}^{(-\frac{{x}^{2}+{y}^{2}}{{\sigma }^{2}})}$$

with ⌠ = λ and *φ* = 0 (even symmetric). Gabors’ spatial frequency (SF) was 2 and 3 cycles/deg (cpd) for MD patients and 3 cpd for controls. A vertical low-contrast Gabor target (Fig. 2) was collinearly flanked, above and below, by two iso-oriented high-contrast Gabors (0.7 Michelson contrast). In addition, a condition with the vertical low-contrast Gabor target flanked by orthogonally oriented Gabors patches was added; with this stimulus configuration, the target detection is not modulated by lateral interactions^28,56^. The contrast threshold of the target was estimated according to a 1 up/3 down staircase. Participants performed a temporal two-alternative forced choice (2AFC). The target was presented in one of the two time intervals, whereas the flankers were always presented in both time intervals. Observers had to report in which time interval the target was presented. Feedback was provided for incorrect trials. Each block was terminated after 120 trials or 16 reversals. Contrast thresholds were estimated by averaging the contrast values corresponding to the last 8 reversals. For the PRL/non-PRL testing, contrast levels from two separated staircases were displayed in a random order over the two different retinal positions.

The two high-contrast collinear flankers were placed at various distances from the target (i.e., 2λ, 3λ, 4λ, and 8λ). The patients were asked to maintain their gaze on the PRL, and stimuli were randomly presented over either the PRL or non-PRL position within a block. Controls had to fixate the center of the screen, and stimuli were randomly presented either left or right of fixation.

#### Visual acuity and crowding stimuli

Visual acuity (eccentric VA) and crowding were measured at the same eccentricity as for the Gabor configuration. Stimuli were generated using Matlab Psychtoolbox^53,54^ and presented at 57 cm. The stimuli were 10 letters (D, N, S, C, K, R, Z, H, O, and V) with Sloan^57^ character type, randomly presented for 133 ms. The target letter was presented randomly at the two eccentricities in the same block, either the PRL/non-PRL for MD patients or 4° left/right from fixation for controls. The size of the letters for measuring acuity threshold and the edge-to-edge spacing for measuring crowding varied according to a psychophysical adaptive procedure (Maximum Likelihood Procedure)^58–60^ that tracked 55% of the participants’ psychometric function within a 60-trial block. The starting streak width was 30 arcmin. Subjects had to verbally report the target letter, and the experimenter registered the answer. The threshold was the values obtained in the last trial.

The crowding stimulus had two different letters vertically flanking the target letter. The streak width of both the target and flanking letters was set 30% higher than the VA threshold obtained at the same eccentricity and the same exposure duration. When tested in the PRL, the MD patients were able to identify all three letters at the largest spacing used (5°). This procedure is often used^34,36,61,62^ to avoid an influence of VA on the measurement of critical spacing for crowding. Crowding was indexed by the critical spacing, defined as the edge-to-edge inter-letter distance at which observers could discriminate the target (the central letter) with 55% accuracy. Differently, from the center-to-center distance, edge-to-edge distance prevents overlay masking of the target by the flankers but has the disadvantage of co-varying with letter acuity (the bigger the target, the larger the center-to-center distance at zero border-to-border distance), thus ultimately underestimating crowding in people with low acuity.

### Procedure

Each subject underwent a testing session of 3 hours in which VA, crowding, and the target contrast thresholds for orthogonal and several collinear configurations were measured monocularly in counterbalanced order.

Contextual influences were estimated by computing the threshold elevation (TE) as:

2 $$TE={\mathrm{log}}_{10}(\frac{CT\_Collinear}{CT\_Orthogonal})$$

where CT_Collinear is the contrast threshold estimated in the collinear condition and CT_Orthogonal is the contrast threshold estimated in the orthogonal condition. TE was calculated separately for each target-to-flanker distance (i.e., 2λ, 3λ, 4λ, and 8λ). We used the orthogonal threshold value obtained at 8λ as a baseline to compute TE (instead of thresholds obtained from the isolated target). The advantage is that the orthogonal configuration still maintains the facilitation induced in the task by the reduced spatial and temporal uncertainty that the presence of the flankers causes so that it is not a confounding factor for the estimation of TE^63^.

### Statistical analysis

Within- and between-group comparisons were carried out with ANOVAs on either contrast threshold or TE using, for pairwise comparisons, t-tests with Bonferroni correction. TE was also analyzed using one-sample, one-tail t-tests based on the hypothesis of TE as either >0 or <0 for suppressive and facilitatory effects, respectively. Visual acuity and crowding data were also analyzed with Tukey tests.

## Results

### Contextual influence results

**Figure 3:**
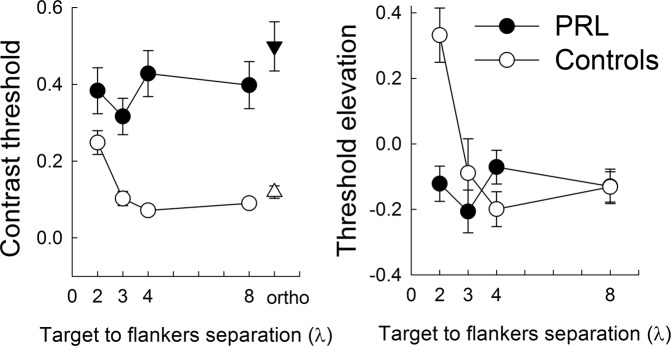
Contrast threshold and TE values (i.e., lateral interaction curves) as a function of target-to-flanker distance for MD patients (PRL data) and controls (data averaged across retinal positions). Contrast thresholds for the orthogonal configuration (8λ) are also shown. Bars indicate standard errors.

Contrast threshold results and TE results are shown in Fig. 3. A mixed-design ANOVA conducted on the contrast threshold data on PRL, including as factors the group (patients vs. controls) and λ (2, 3, 4, and 8λ), revealed that thresholds were significantly higher for the MD patients (F(1,18) = 13.7, p = 0.002, partial-η2 = 0.422). The effect of λ was significant (F(3,54) = 3.308, p = 0.027, partial-η2 = 0.155). The interaction group x λ was also significant (F(3,54) = 4.024, p = 0.012, partial-η2 = 0.183). Pairwise comparisons revealed a group difference for the 3 λ (p = 0.005), 4 λ (p < 0.001), and 8 λ (p = 0.002) and higher thresholds at 2 λ compared to 3 λ (p = 0.026), 4 λ (p = 0.039). None of the differences in thresholds across λs were significant in the MD group.

A mixed-design ANOVA conducted on TE data, including as factors the group (patients vs. controls) and the λ (2, 3, 4, and 8λ) indicated that the effect of group was not significant (F(1,18) = 2.752, p = 0.114, partial-η2 = 0.133), while both the effect of λ (F(3,54) = 10.09, p < 0.001, partial-η2 = 0.359), and the group x λ interaction (F(3,54) = 10.561, p < 0.001, partial-η2 = 0.370) were. Post hoc comparisons showed higher TE for the smallest λ compared to others (2 vs. 3λ, p = 0.002; 2 vs. 4λ, p < 0.001; 2 vs. 8λ, p = 0.009). Most importantly, there was a significant group difference at the smallest λ only (p < 0.001), and a different effect of the smallest λ in the two groups: For controls, TEs were higher at the smallest λ compared to the others (2 vs. 3λ, p = 0.001; 2 vs. 4λ, p < 0.001; 2 vs. 8λ, p = 0.002); for the MD group, there was not any difference in TE between the different λs (p = 0.99). Since TE > reflects inhibitory lateral interaction and TE <0 facilitatory, one-sample, one-tail t-tests were conducted to test the null hypothesis of 0 TE. As expected TE resulted generally negative for λs ≥3 for for both MD patients (3λ: (t(12) = −3.2, p = 0.004; 4λ: t(12) = −1.4, p = 0.096; 8λ: t(12) = −2.47, p = 0.014) and controls (3λ: t(6) = −0.85, p = 0.21; 4λ: t(6) = −3.74, p = 0.005; 8λ: t(6) = −2.8, p = 0.015)), confirming that contextual enhancement occurs at larger λs in the periphery than in the fovea^31,33,41^. At the shortest λ, TE assumed, as expected, positive values for controls (t(6) = 4.01, p = 0.003); however, at the same shortest λ the values of TE for MD patients were negative (t(12) = −2.3, p = 0.02).

**Figure 4:**
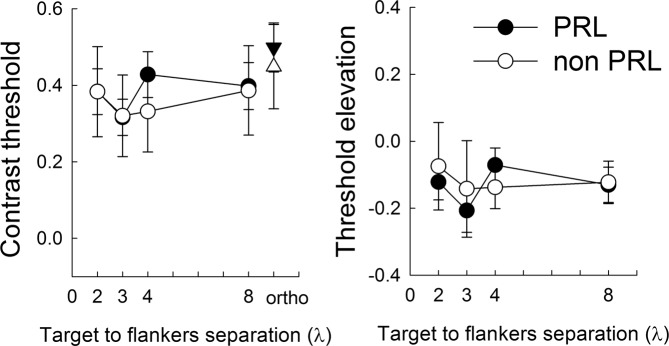
Contrast threshold and TE values (i.e., lateral interaction curves) as a function of target-to-flanker distance for 8 MD subjects in the PRL and non-PRL retinal positions. Contrast thresholds for the orthogonal configuration (8λ) are also shown. Bars indicate standard errors.

Comparison of contrast thresholds and TE data obtained in the PRL and non-PRL of MD patients are shown in Fig. 4. Repeated-measures ANOVAs, including as factors the retinal locus (PRL and non-PRL) and the λ (2, 3, 4, and 8λ), were conducted on both the contrast thresholds and TE data of the subgroup of 8 patients that were tested with stimuli presented in both the PRL and non-PRL positions.

The ANOVA on contrast threshold revealed that neither the main factors (PRL: F(1,19) = 0.077, p = 0.785, partial-η2 = 0.004; λ: F(3,57) = 0.947, p = 0.424, partial-η2 = 0.047) nor the PRL x λ interaction (F(3,57) = 0.463, p = 0.709, partial-η2 = 0.024) were significant. Similarly, the ANOVA on TE data did not reveal a significant effect of the retinal locus (F(1,19) = 0.027, p = 0.872, partial-η2 = 0.001), of λ (F(3,57) = 0.546, p = 0.653, partial-η2 = 0.028), and of the interaction between PRL and λ (F(3,57) = 0.389, p = 0.762, partial-η2 = 0.02).

### Acuity and crowding results

**Figure 5:**
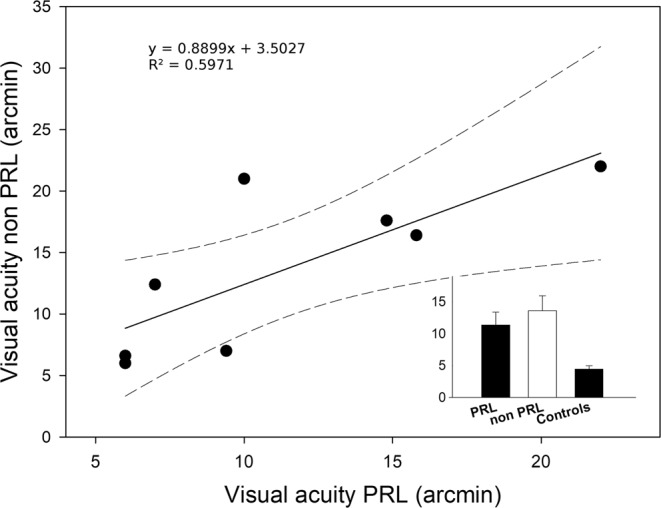
Scatter plot represents visual acuity obtained with stimuli presented at the non-PRL regressed on visual acuity obtained with stimuli presented at the PRL only for the patients for which the two positions were symmetrical with respect to the fovea. Dashed lines represent 95% confidence intervals. Average visual acuity for this subgroup (n = 8), and for the control group (n = 7) is also shown in the bar chart.

The visual acuity of the 8 patients who had a reliable measure in both the PRL and non-PRL are shown in Fig. 5. The one-way ANOVA conducted on these data, with group as a factor (controls, PRL, non-PRL) showed a significant effect of group (F(2,20) = 6.22, p = 0.007, partial-η2 = 0.384), indicating higher acuity for controls than MD patients both when tested at the PRL (difference = 6.917, p = 0.046) and non-PRL positions (difference = 9.167, p = 0.007). The difference between PRL and non-PRL was not significant (difference = −2.25, p = 0.67). Even considering the small sample and the high variability in VA data, this lack of difference confirms that development of a PRL is not strictly linked to an advantage in terms of visual acuity over the other retinal quadrants^64,65^. Moreover, the two acuity values for the 8 patients are highly correlated (R = 0.78, p = 0.022).

**Figure 6:**
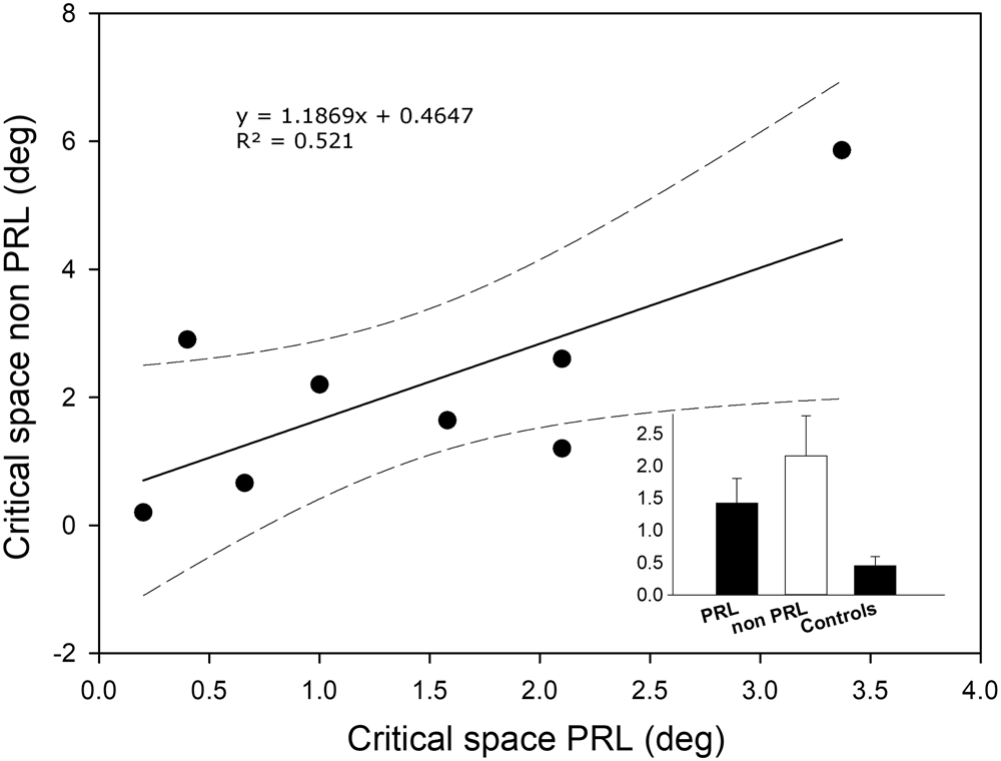
Scatter plot represents critical spacing for crowding obtained with stimuli presented at the non-PRL regressed on critical spacing for crowding obtained with stimuli presented at the PRL only for the patients for which the two positions were symmetrical with respect to the fovea. Dashed lines represent 95% confidence intervals. Average critical spacing for crowding for this subgroup (n = 8), and for the control group (n = 7) is also shown in the bar chart.

Individual crowding data of the 8 patients who had a reliable measure in both the PRL and non-PRL are shown in Fig. 6. The one-way ANOVA conducted on these data revealed a significant effect of group (F(2,20) = 3.516, p = 0.049, partial-η2 = 0.26). Post hoc comparison showed a significant difference between controls and MD patients when tested at the non-PRL (difference = 1.693, p = 0.039) but not when tested at the PRL (difference = 0.962, p = 0.3). The difference between PRL and non-PRL was not significant (difference = −0.731, p = 0.47). Visual acuity and crowding measures should be independent by definition. We checked this assumption by calculating the correlation between the two measures. We found that the negative correlation did not reach significance (R = −0.35, p = 0.18) despite the fact that the operative definition of critical spacing as the edge-to-edge inter-letter distance may have inflated this estimate (see Method section).

## Discussion

Using a two-interval forced choice task, contrast threshold for a low-contrast target Gabor flanked by two collinear high-contrast Gabors presented at eccentricities varying between 3° and 8° was measured in a group of subjects with MD and in an age-matched control group. Target-to-flanker separation, varied in terms of the Gabor’s carrier wavelength unit (λ), was 2λ, 3λ, 4λ, and 8λ. The contextual influence of the flankers was quantified by the threshold modulation (TE), indicating the change in contrast threshold obtained at each of the four λs, relative to the baseline condition with no contextual influence (orthogonal flankers, 8λ).

Results showed contextual enhancement at λs higher than in the fovea (4-8λ). At 2λ, all controls had inhibition. Only 2 of the patients had inhibition, 2 had a TE close to zero and 9 of them had negative TE, indicating facilitatory contextual influences. This change in contextual influences at the shortest λ in MD patients was associated, both at the PRL and non-PRL, with an increase of contrast threshold for the target, as well as with reduced visual acuity and a larger crowding effect. Is the switch between inhibition and facilitation at the shortest λ an index of cortical plasticity or it could be explained by the same model used to interpret pshychophysical data from normal viewers? An answer to this question comes from establishing whether TE in MDs is well described by the variation of TE as a function of flanker/target contrast ratio in normal vision^31,66–69^. Zenger & Sagi (1996) proposed a model for contextual influences in which:

i. At a close distance, the sensitivity to a low contrast target is reduced by the presence of high contrast flankers.
ii. When the contrast of target increases the reduction in sensitivity progressively decreases and then turns into facilitation. The switch happens when the contrast of the target is still lower (around three times) with respect to that of the flankers.
iii. When the contrast of target approaches that of the flankers there is a deep in the facilitation.
iv. However,when the contrast of target surpass that of the flankers the facilitatory effect progressively reduces and then disappear.

**Figure 7:**
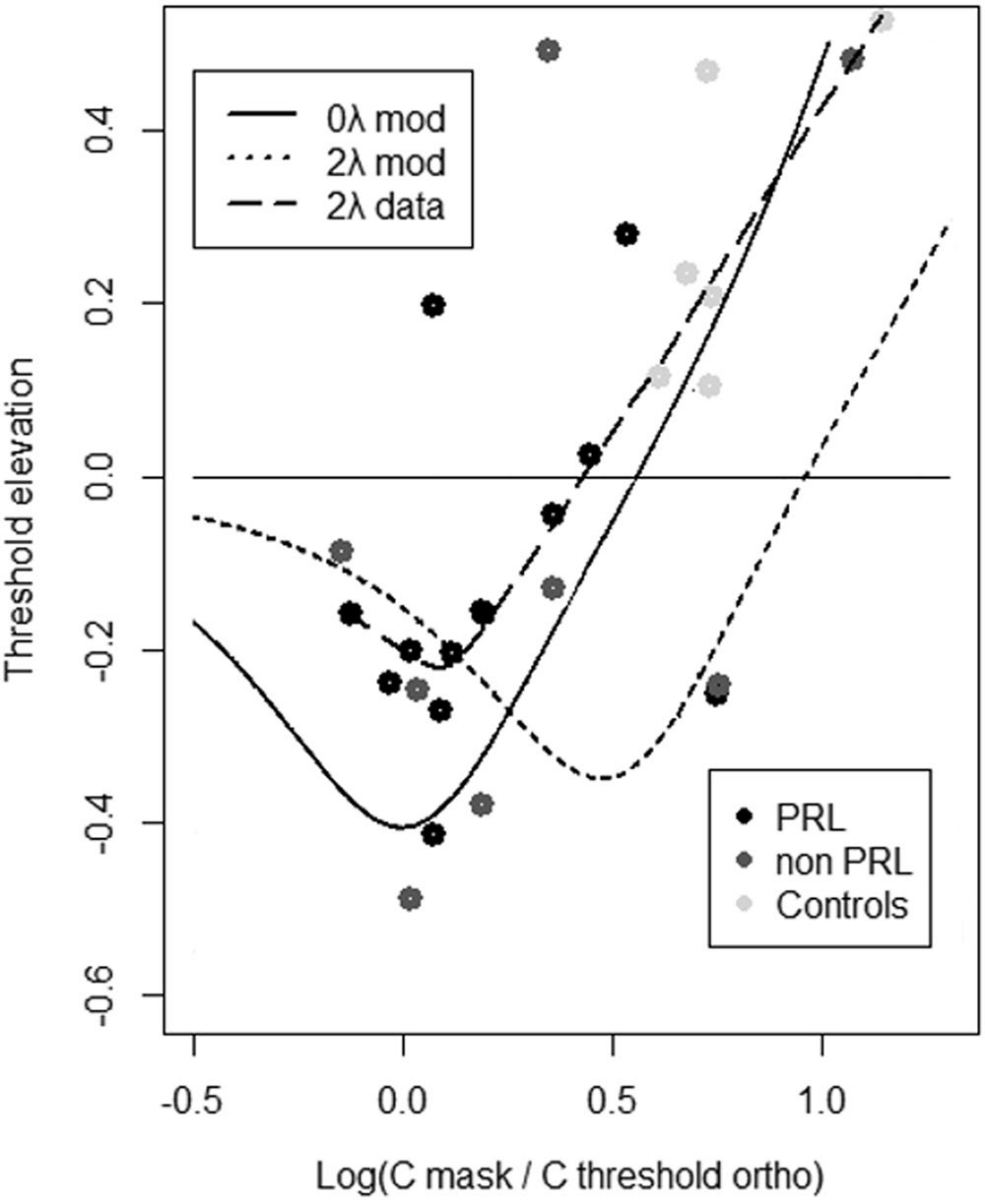
TEs as a function of log _10_(Contrast_flankers_/Contrast _target threshold_) in the 8λ orthogonal condition are shown for the pooled data obtained by patients at the PRL (n = 13) and at the non-PRL (n = 8), and for the data of the control group (N = 7). Prediction for the 0 λ and 2λ based on the model from Zenger and Sagi (1996) are shown together with the locally weighted scatterplot smoothing (lowess) from our data.

The model postulates a contrast dependent modulation of the contextual effect of the flankers that shift s progressively from inhibition to facilitation depending on flanker/target contrast ratio. We verified this by pulling the data of the two groups toghether and regressing the TE at 2λ as a function of log 10(Contrast_flankers_/Contrast _target threshold_) in the 8λ orthogonal condition. We performed a locally-weighted polynomial regression (lowess)^70,71^ in R^72^ with a 50% smoothing span that leads to an R^2^ of 0.52 (correlation between raw and estimated data). Finally, we superimposed raw data and fit line over the model predictions from Zenger and Sagi (1996) at 0λ and 2λ. As the Fig. 7 shows, the regression line derived form our dataset approximate very vell the one predicted by the model, in particular the line that refers to the 0 λ, as expected by the fact that increasing eccentricity would shift the curve leftwards. Thus, the transition from inhibition to facilitation that most MD patients show at high contrast threshold suggests improved efficiency in integrating/grouping elements, possibly mediated by an integration between the flanker and target within the 2^nd^ order integrative field^31^.

Our results do not support the hypothesis that MD present cortical reorganization leading to a use-dependent increase of long-range connectivity. In this case, with the low contrast target, we would have obtained increased contrast enhancement at the range of target-to-flanker distances at which facilitation occurs in normal vision. In the previous literature, the reduced collinear inhibition found in MD has been interpreted as a sign of neural plasticity, linked with a change in receptive field size^35,49^. We shed new light on this phenomenon and propose a different interpretation. If we consider together all our participants including the controls, our data show that the reduction in collinear inhibition and the switch towards facilitation are clearly linked with the baseline contrast sensitivity of the single subject in the orthogonal configuration. This switch can be well described by the same model previously proposed for the normal vision^31^ and thus our results support the hypothesis that inhibitory target-flanker separations (short) become facilitatory in MD as it would do in controls if their contrast threshold were 4-5 fold higher. Taking all this information into consideration, the reduction of inhibition cannot be ascribed to neural plasticity in PRL but must be considered as a by-product of the same retinal degeneration that may deplete the patient’s vision at the boundary of the scotoma.

To conclude, our result is that a reduction of contrast gain at the boundary of the scotoma is associated not only to reduced resolution^73^, which indeed MDs show for stimuli presented at the boundary of the scotoma, but also to a change in the way neighboring elements are integrated.

## Data Availability

The datasets analyzed during the current study are available at the following repository: 10.17632/7n6zhxh37s.1.
